# Berberine Alleviates Intestinal Inflammation by Disrupting Pathological Macrophage–Epithelial Crosstalk in Macrophage–Organoid Co-Culture Model

**DOI:** 10.3390/ijms262010161

**Published:** 2025-10-19

**Authors:** Yuncong Han, Mengting Li, Tian Chen, Chen Wang, Hong Zhou, Tunan Zhou, Runqing Jia, Ying Chen, Qin Hu

**Affiliations:** 1 College of Chemistry and Life Science, Beijing University of Technology, Beijing 100124, China; hanyuncong@emails.bjut.edu.cn (Y.H.); chentian@emails.bjut.edu.cn (T.C.); wangchen202484048@emails.bjut.edu.cn (C.W.); zhouhong@emails.bjut.edu.cn (H.Z.); z969081067@emails.bjut.edu.cn (T.Z.); 2 Institute of Chinese Materia Medica, China Academy of Chinese Medical Sciences, Beijing 100700, China; lmthahaha@outlook.com (M.L.); ychen@icmm.ac.cn (Y.C.)

**Keywords:** berberine, intestinal organoid, co-culture model, anti-inflammation

## Abstract

Berberine (BBR), a benzylisoquinoline alkaloid isolated from Chinese herb *Coptis chinensis*, has been widely used clinically to treat intestinal infectious diseases. Recently, it has been found to have multiple pharmacological effects, including anti-inflammatory activity and immune effects in inflammatory bowel disease (IBD). However, its exact targets remain to be elucidated. In this study, we used a mouse intestinal organoid–macrophage co-culture model to investigate the anti-inflammatory effects and immune effects of BBR. Our findings demonstrated that lipopolysaccharide (LPS) induced more robust inflammatory responses and epithelium damage in the co-culture system compared to the organoid alone. BBR effectively attenuated inflammation and restored epithelial barrier integrity by suppressing M1 macrophage polarisation and infiltration, alongside upregulating the expression and organisation of tight junction protein zonula occludens-1 (ZO-1). RNA sequencing and proteomic analysis revealed that BBR disrupted organoid–macrophage interaction by inhibiting chemokine (e.g., C-X-C motif chemokine ligand 1 (CXCL1) and macrophage migration inhibitory factor (MIF)) release from epithelial cells, thereby reducing macrophage recruitment. Collectively, our study establishes the organoid–macrophage co-culture system as a more physiologically relevant model for studying epithelial–immune interactions and elucidates the multi-target mechanism of BBR, which concurrently modulates epithelial cells, macrophages, and their crosstalk. These findings lay the foundation for further exploration of the therapeutic potential of BBR in inflammatory bowel disease and the development of targeted therapies that regulate cell interactions.

## 1. Introduction

Berberine (BBR) is a benzylisoquinoline alkaloid derived from traditional Chinese herbs such as *Coptis chinensis* and *Phellodendron chinensis*. It has long been used for treating gastrointestinal diseases including diarrhoea and abdominal pain [1]. Growing evidence demonstrates that berberine possesses various therapeutic potentials, exhibiting antibacterial, antiviral, anti-inflammatory, immunomodulatory, metabolic regulatory, and antitumor effects [2]. These varied biological activities have broadened its clinical application from traditional over-the-counter antidiarrheal agents to cardiovascular diseases, metabolic syndromes, central nervous system disorders, and tumours [3,4].

Recent clinical data and trials have highlighted the therapeutic efficacy of BBR in the management of various intestinal disorders, such as intestinal infections, inflammatory bowel disease (IBD), and intestinal tumours, although the underlying mechanisms are not yet fully elucidated. In multiple preclinical intestinal disease models, including dextran sulphate sodium/trinitrobenzene sulfonic acid (DSS/TNBS)-induced ulcerative colitis, cecal ligation and puncture (CLP)-mediated intestinal injury, azoxymethane/dextran sulphate sodium (AOM/DSS)-induced colorectal carcinogenesis, irritable bowel syndrome (IBS)-like rodent models, and lipopolysaccharide (LPS)-induced sepsis models [5,6,7], BBR has consistently alleviated pathological conditions. These effects include recovering the intestinal epithelial barrier, restoring crypt architecture, reducing neutrophil and macrophage infiltration, and modulating immune cell populations [8].

BBR’s well-established antimicrobial activity significantly modulates intestinal microecology by selectively altering microbiota composition (e.g., enriching *Bifidobacterium* populations) and regulating the release of metabolic byproducts such as short-chain fatty acids [9]. Its anti-inflammatory and antioxidant properties have also been extensively studied in immune cells. BBR effectively attenuates pro-inflammatory M1 macrophage polarisation [10], suppresses innate lymphoid cells (ILC)1, and increases ILC3 proportion, thereby rebalancing the ILC repertoire. Additionally, BBR modulates CD4 T cell differentiation into regulatory T cells (Treg), regulating the Treg/Th17 balance, and reducing the ratio of interferon-gamma (IFNγ) producing CD4 T cells in the lamina propria [11]. Furthermore, BBR reduces pro-inflammatory cytokines (interleukin 17 (IL-17) and tumour necrosis factor-α (TNF-α)) in tissues and serum, increases interleukin-22 (IL-22) expression, and inhibits NOD-like receptor thermal protein domain-associated protein 3 (NLRP3) inflammasome activation [12]. These immune modulatory effects involve inhibiting several key signalling pathways, including the phosphatidylinositol 3-kinase (PI3K)/protein kinase B (AKT)/mammalian target of rapamycin (mTOR) pathway, the mitogen-activated protein kinase (MAPK)/extracellular signal-regulated kinase (ERK) pathway, the c-Jun N-terminal kinase (JNK)/signal transducer and activator of transcription (STAT) pathway, and nuclear factor kappa B (NF-κB) signalling [13,14,15]. In addition to immune modulation, the BBR protects the intestinal epithelial barrier by reducing cell apoptosis via AMP-activated protein kinase (AMPK) pathway activation [16] and restoring tight junction integrity [17]. It also alleviates oxidative damage through reactive oxygen species (ROS) scavenging and the Nrf2-mediated induction of antioxidant enzymes [18], thereby promoting redox homeostasis. Recent findings reveal that BBR facilitates mucosal healing and barrier regeneration by regulating Wnt/β-catenin signalling and bitter taste receptors on Tuft cells [19,20].

Despite acting as a multi-target drug with intricate regulatory mechanisms, BBR’s specific effects on the intestinal microenvironment remain incompletely characterised. The complexity of microbiota–host interactions introduces significant variables for in vivo studies exploring BBR’s impact on particular cellular interactions. To address this, we employed murine intestine organoid model in our study. First established in 2009 [21], these three-dimensional, self-organising organoids were created from intestinal leucine-rich repeat-containing g-protein-coupled receptor 5 (Lgr5+) stem cells in vitro, which recapitulate epithelium containing stem cells, Paneth cells, goblet cells, and enterocytes. These spontaneously assembled 3D structures effectively simulate crypt–villus organisation and barrier function critical for inflammatory processes [22], making them crucial tools for investigating gastrointestinal pathophysiology [23].

In the present study, we developed an intestinal–immune crosstalk model through macrophage–organoid Transwell co-culture systems. We used LPS to induce inflammation and tissue damage. Through this model, we investigated the immunomodulatory and epithelial barrier protective effects of BBR, and elucidated how BBR orchestrates intestinal microenvironmental homeostasis via immune-epithelial interactions.

## 2. Results

### 2.1. BBR Antagonised LPS-Induced Inflammation on Murine Intestinal Organoids

To assess the cytotoxicity of BBR in murine intestinal organoids, cell viability was evaluated using ATP bioluminescence and Edu cell proliferation assay. BBR exhibited an IC_50_ of 17.09 μM according to Edu quantification (Figure S1A), and the BBR at concentration of 0.3–3 μM showed no significant effect on cellular ATP activity. These non-cytotoxic doses were subsequently used.

**Figure 1:**
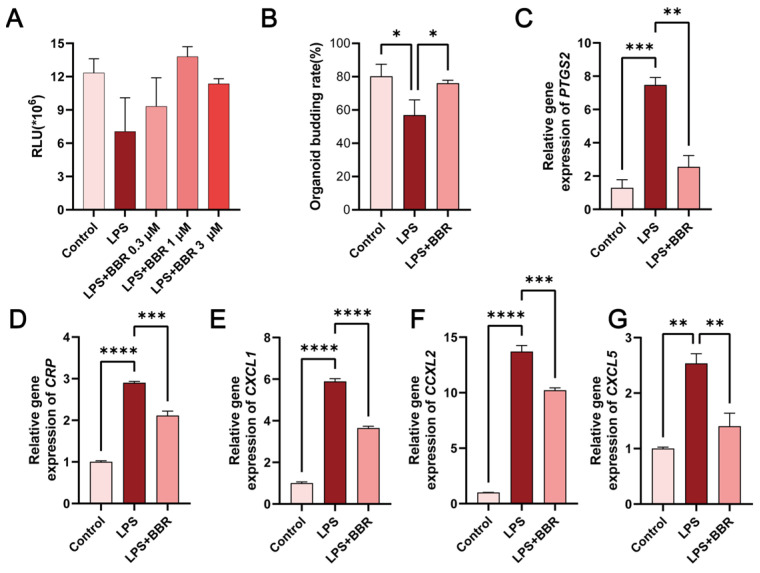
Berberine (BBR) is protective against lipopolysaccharide (LPS)-induced inflammatory injury in intestinal organoids. (**A**) 50 μg/mL of LPS and 0.3–3 μM of BBR were applied to intestinal organoids and treated for 24 h. Cell viability was detected using the CellTiter-Glo method. Overall, 50 μg/mL of LPS and 1 μM of BBR were applied to intestinal organoids and treated for 24 h. (**B**) Budding rates of at least 150 organoids were counted using ImageJ software. Expression of *prostaglandin-endoperoxide synthase 2* (*PTGS2*) (**C**), *c-reactive protein* (*CRP*) (**D**), *C-X-C motif chemokine ligand 1* (*CXCL1*) (**E**), *C-X-C motif chemokine ligand 2* (*CXCL2*) (**F**), and *C-X-C motif chemokine ligand 5* (*CXCL5*) (**G**) pro-inflammatory cytokines was verified by qPCR. Values given are the mean and standard deviation of triplicate replicates. All experiments were performed three times. **** *p* < 0.0001, *** *p* < 0.001, ** *p* < 0.01, * *p* < 0.05 vs. the LPS group.

The treatment of LPS (50 μg/mL) for 24 h reduced cell viability (Figure 1A) and significantly decreased the budding rates (Figure 1B) of cultured intestinal organoid. LPS also increased membrane permeability, as measured by fluorescein isothiocyanate (FITC) dextran (4KD) flux assay (Figure S2), and markedly upregulated pro-inflammatory genes, including *prostaglandin-endoperoxide synthase 2* (*PTGS2*) (Figure 1C), *c-reactive protein* (*CRP*) (Figure 1D), *C-X-C motif chemokine ligand 1* (*CXCL1*) (Figure 1E), *C-X-C motif chemokine ligand 2* (*CXCL2*) (Figure 1F), and *C-X-C motif chemokine ligand 5* (*CXCL5*) (Figure 1G). BBR treatment significantly restored both organoid viability and budding rate, while significantly suppressing cytokine over-expression. These results suggest that BBR has a protective effect against LPS-induced inflammatory damage to intestinal organoid.

### 2.2. BBR Has Anti-Inflammatory and Barrier-Protective Effects in a Macrophage–Intestinal Organoids Co-Culture Model

**Figure 2:**
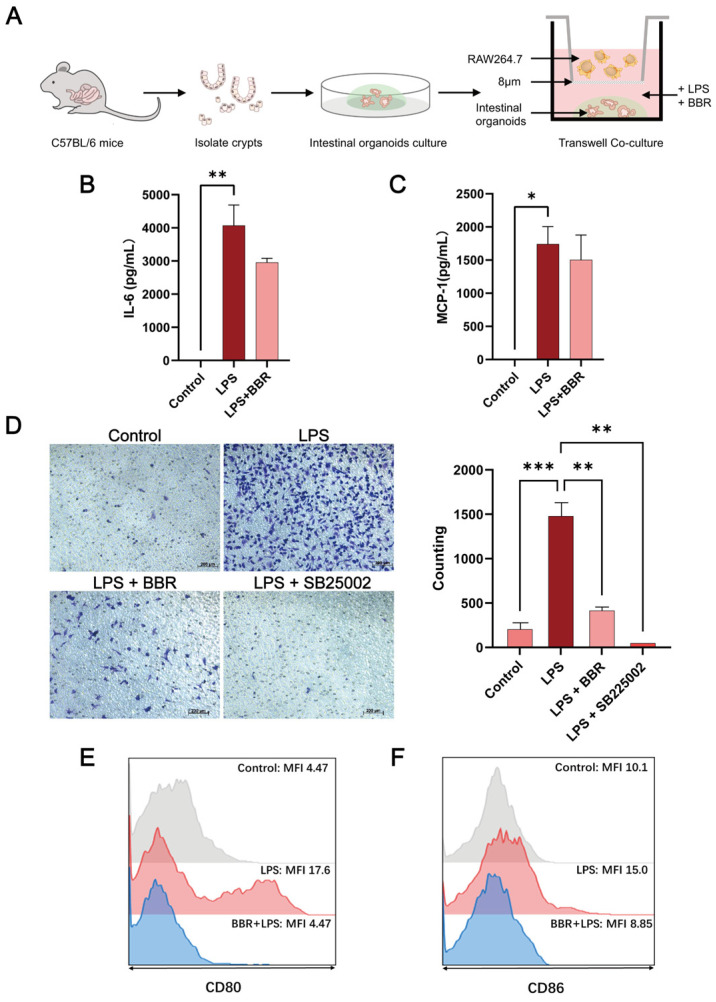
BBR antagonises macrophage chemotaxis and M1 polarisation capacity in inflammation. (**A**) Schematic of Transwell co-culture of intestinal organoid and RAW264.7 cells. Overall, 50 μg/mL of LPS and 1 μM of BBR were applied to the lower chamber of the co-culture system and treated for 24 h. Changes in the expression of inflammatory factors interleukin-6 (IL-6) (**B**) and monocyte chemoattractant protein-1 (MCP-1) (**C**) were detected using the enzyme-linked immunosorbent assay. (**D**) After 50 μg/mL of LPS and 50 nM of SB225002 were applied as a positive control, migrating cells were counted in five randomly selected fields of view using 0.1% crystal violet staining to detect the migration efficiency of RAW264.7 cells. The median fluorescence intensity (MFI) of CD80 (**E**) and CD86 (**F**) in RAW264.7 cells in the co-culture system was detected using flow cytometry. The values given are the mean and standard deviation of three replicates. All experiments were performed three times. *** *p* < 0.001, ** *p* < 0.01, * *p* < 0.05 vs. the LPS group.

To investigate interactions between intestinal organoids and immune cells, a Transwell co-culture system (8 μm pore, Figure 2A) was used with intestinal organoids cultured in the lower chamber and RAW264.7 cells seeded in the upper chamber. LPS and BBR were added to the intestinal organoid culture for 24 h. Analysis of the secretion of pro-inflamatory cytokines showed that LPS significantly induced an increase in the expression of interleukin-6 (IL-6) (Figure 2B) and monocyte chemoattractant protein-1 (MCP-1) (Figure 2C). It also significantly enhanced the migration of RAW264.7 cells (Figure 2D), which was attenuated with the C-X-C chemokine receptor type 2 (CXCR2) inhibitor SB225002 and BBR treatment, suggesting that BBR can effectively suppress macrophage recruitment. Flow cytometry analysis revealed that LPS significantly induced M1 polarisation markers CD80 (Figure 2E) and CD86 (Figure 2F); BBR treatment antagonised this M1 polarisation, confirming its ability to disrupt macrophage chemotaxis and pro-inflammatory polarisation.

**Figure 3:**
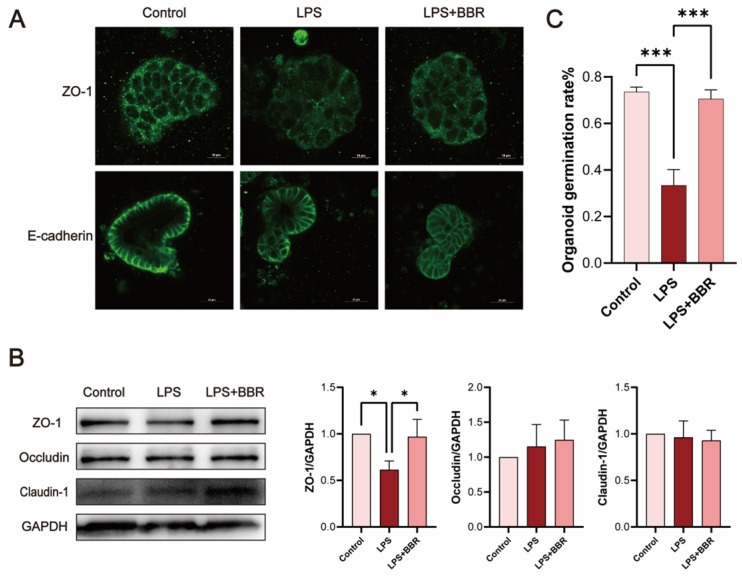
BBR repairs inflammation-induced barrier damage and reduces pro-inflammatory factor secretion. Overall, 50 μg/mL of LPS and 1 μM of BBR were applied to the lower chamber of the co-culture system and treated for 24 h. (**A**) Localisation of zonula occludens-1 (ZO-1) and E-cadherin was detected by immunofluorescence. Scale bar of ZO-1, 10 μm. Scale bar of E-cadherin, 25 μm. (**B**) The expression of ZO-1, claudin-1, and occludin proteins was detected by Western blot. (**C**) Budding rates of at least 150 organoids were counted using ImageJ software. Values given are the mean and standard deviation of three replicates. All experiments were performed two–three times. *** *p* < 0.001, * *p* < 0.05 vs. the LPS group.

Immunofluorescence analysis (Figure 3A) revealed that after LPS treatment, the smooth, continuous expression of tight junction (TJ) protein zonula occludens-1 (ZO-1) became blurred and partially broken, which BBR restored to control levels. Western blot analysis confirmed that ZO-1 protein expression was reduced by LPS and rescued by BBR treatment (Figure 3B). No significant alterations were observed in E-cadherin localisation or claudin-1/occludin expression. Furthermore, LPS treatment markedly reduced the budding rate of organoids, which were rescued by BBR (Figure 3C).

Collectively, these findings suggest that in the macrophage–intestinal organoid co-culture model, BBR significantly inhibited the M1 polarisation/chemotaxis and preserved the viability and integrity of intestinal epithelial barrier structure.

### 2.3. BBR Regulated Epithelial–Macrophage Interaction

**Figure 4:**
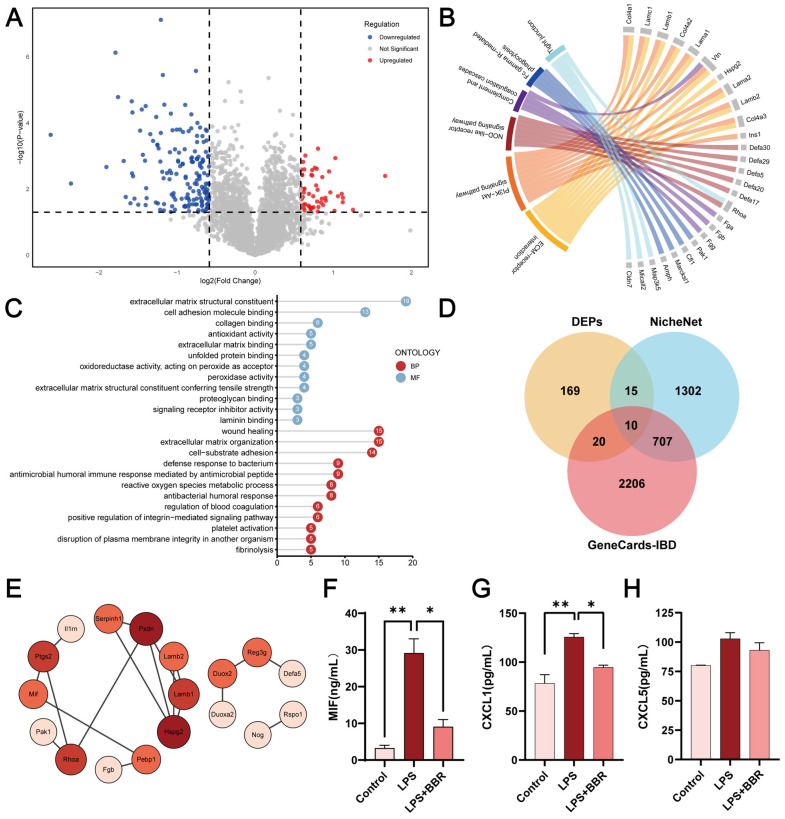
Proteomic analysis of the effect of BBR on intestinal organoid in inflammation. Overall, 50 μg/mL of LPS and 1 μM of BBR were applied to the intestinal organoids and treated for 24 h. The proteins were collected and analysed by proteomics. The 214 differentially expressed proteins (DEPs) obtained from the comparison of the LPS and BBR-treated groups were plotted as volcano plot (**A**) and analysed for Kyoto Encyclopedia of Genes and Genomes (KEGG) (**B**) and Gene Ontology (GO) (**C**) enrichment. A Venn diagram analysis (**D**) was performed on the set of DEPs obtained from proteomics analyses, and the set of intercellular communication genes obtained from the NicheNet analysis tool, and the set of inflammatory bowel disease (IBD) related genes based on the GeneCards database. (**E**) Protein interaction networks at the intersection of DEPs and IBD gene sets were identified using the search tool for retrieval of interacting genes (STRING) database. Changes in the expression of inflammatory factors migration inhibitory factor (MIF) (**F**), CXCL1 (**G**) and CXCL5 (**H**) were detected using the enzyme-linked immunosorbent assay. The values given are the mean and standard deviation of three replicates. The experiment was performed in triplicate and repeated 3–4 times. ** *p* < 0.01, * *p* < 0.05 vs. the LPS group.

Proteomic analysis of intestinal organoids was conducted to analyse the differentially expressed proteins (DEPs), with Foldchange > 1.5 and *p* < 0.05 as the screening criteria. Compared to the control group, LPS-induced the upregulation of 99 proteins and downregulation of 69 proteins (Figure S3A). Compared to the LPS group, BBR significantly upregulated 53 proteins and downregulated 161 proteins (Figure 4A). Kyoto Encyclopedia of Genes and Genomes (KEGG) and Gene Ontology (GO) enrichment analysis linked LPS-induced DEPs to pathways such as innate immune response activation and antigen processing/presentation (Figure S3B,C), while BBR-modulated DEPs were significantly enriched in pathways including positive regulation of reactive oxygen species (ROS) metabolic process, NOD-like receptor (NLR) signalling, and antibacterial process (Figure 4B,C).

Venn diagram analysis against NicheNet database identified 25 cell–cell interaction proteins within the DEP set (e.g., migration inhibitory factor (MIF), PTGS2) (Figure 4D). GO and KEGG enrichment analysis linked these DEPs to the activation of the MAPK signalling, ERK cascade, and pathways related to cell migration and proliferation (Figure S3D). The comparison of the DEPs with the IBD GeneCards dataset (2943 genes) revealed 30 IBD-associated proteins (Figure S3E), showing enrichment for oxidative stress response, and C-type lectin receptor (CLR) signalling pathways. Search tool for retrieval of interacting genes (STRING)-based protein–protein interaction (PPI) network highlighted key BBR-modulated nodes including interleukin-1 receptor antagonist (IL-1RN)-PTGS2-MIF (Figure 4E). Overlap analysis further identified 10 hub proteins (MIF, IL1RN, claudin 7 (CLDN7), PTGS2) shared between DEP-IBD gene set and DEP-NicheNet datasets, suggesting BBR’s potential role in modulating epithelial–macrophage crosstalk. Supporting this, ELISA of MIF (Figure 4F), CXCL1 (Figure 4G) and CXCL5 (Figure 4H) demonstrated that LPS significantly increased the secretion of CXCL1 and MIF, which were attenuated by BBR. Molecular docking analysis further indicated strong binding affinity between BBR and MIF (−7.8 kcal/mol) (Figure S3F).

In summary, BBR modulated interactions between epithelial and macrophages, particularly via chemokines such as MIF, to regulate macrophage chemotaxis.

### 2.4. Macrophage–Intestinal Organoids Co-Culture Exhibits a More Robust Inflammatory Microenvironment

**Figure 5:**
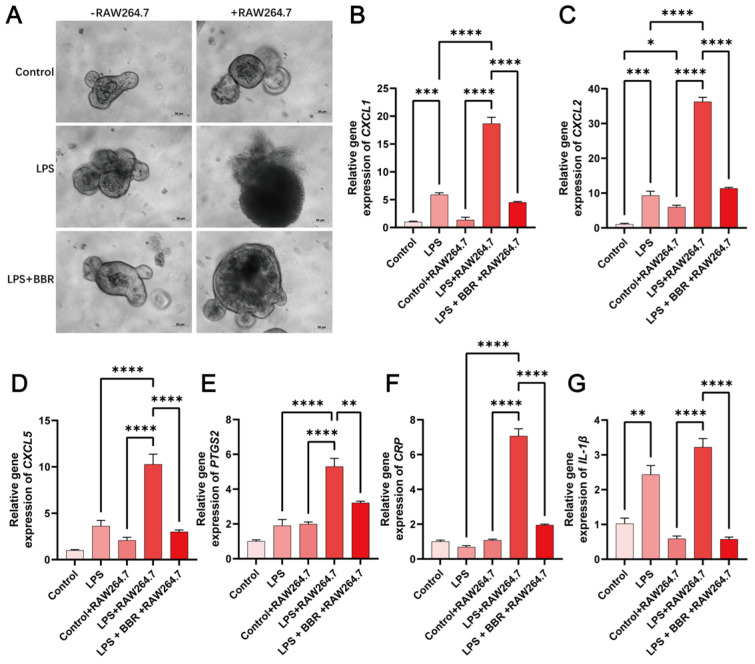
BBR modulates epithelial barrier and macrophage interactions during inflammation. Overall, 50 μg/mL of LPS and 1 μM of BBR were applied to the lower chamber of the co-culture system and treated for 24 h. (**A**) Representative images of organoids taken using an inverted microscope. Scale bar, 50 μm. Expression of *CXCL1* (**B**), *CXCL2* (**C**), *CXCL5* (**D**), *PTGS2* (**E**), *CRP* (**F**), and *interleukin-1β* (*IL-1β*) (**G**) pro-inflammatory cytokines were verified by qPCR. Values given are the mean and standard deviation of three replicates. All experiments were performed three times. **** *p* < 0.0001, *** *p* < 0.001, ** *p* < 0.01 vs. the LPS group or the LPS+RAW264.7. * *p* < 0.05 vs. the control + RAW264.7.

Morphometric analysis revealed that LPS alone induced minimal structural disruption in murine intestinal organoids (Figure 5A), whereas when RAW264.7 macrophages were introduced, LPS triggered pronounced epithelial death. BBR could alleviate coculture-enhanced toxicity and restore cell morphology. RT-PCR quantification of pro-inflammatory genes confirmed macrophage-dependent amplification of LPS-driven inflammation. BBR significantly attenuated LPS-induced expression in *CXCL1* (Figure 5B), *CXCL2* (Figure 5C), *CXCL5* (Figure 5D), *PTGS2* (Figure 5E), *CRP* (Figure 5F) and *interleukin-1β* (*IL-1β*) (Figure 5G), suggesting its efficacy in blocking macrophage–epithelium interactions.

**Figure 6:**
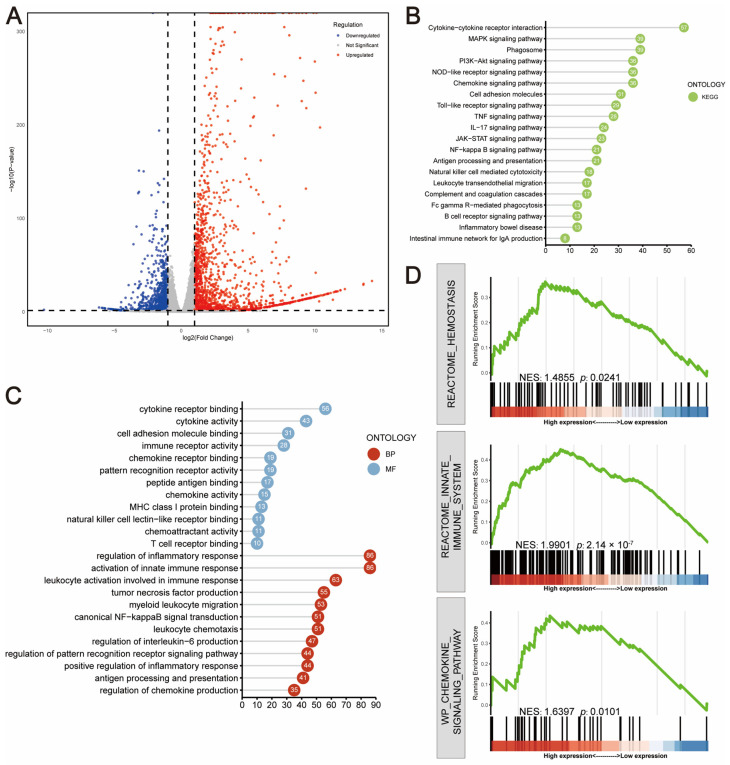
Transcriptomic analyses showed that co-culture amplifies LPS-induced inflammation. Overall, 50 μg/mL of LPS was applied to the intestinal organoids in the lower chamber of the co-culture system and treated for 24 h. Extracted RNA from the intestinal organoids was collected and analysed for transcriptomics. The 1176 differentially expressed genes (DEGs) obtained from transcriptomics analysis were mapped as volcano plots (**A**) and subjected to KEGG (**B**) and GO (**C**) enrichment analyses. Gene set enrichment analysis (GSEA) analyses showed that DEGs were significantly enriched and upregulated in pathways such as innate immunity, chemokine signalling pathway, and haemostasis (**D**). The experiment was performed in triplicate and repeated 3–4 times.

RNA-seq was then used to identify differentially expressed genes (DEGs) modulated by LPS and BBR with the presence of RAW264.7 cells. The data revealed that co-culture with macrophage significantly amplified the LPS-induced inflammatory response, presented by 1176 DEGs (Figure 6A). KEGG enrichment (Figure 6B) and GO pathway analysis (Figure 6C) showed that these genes were mainly enriched in functions related to the regulation of innate immune response and regulation of inflammatory response. Gene set enrichment analysis (GSEA) [24,25] further demonstrated the significant enrichment of upregulation of IFN response pathways in LPS-treated intestinal organoids upon RAW cell involvement (Figure 6D).

**Figure 7:**
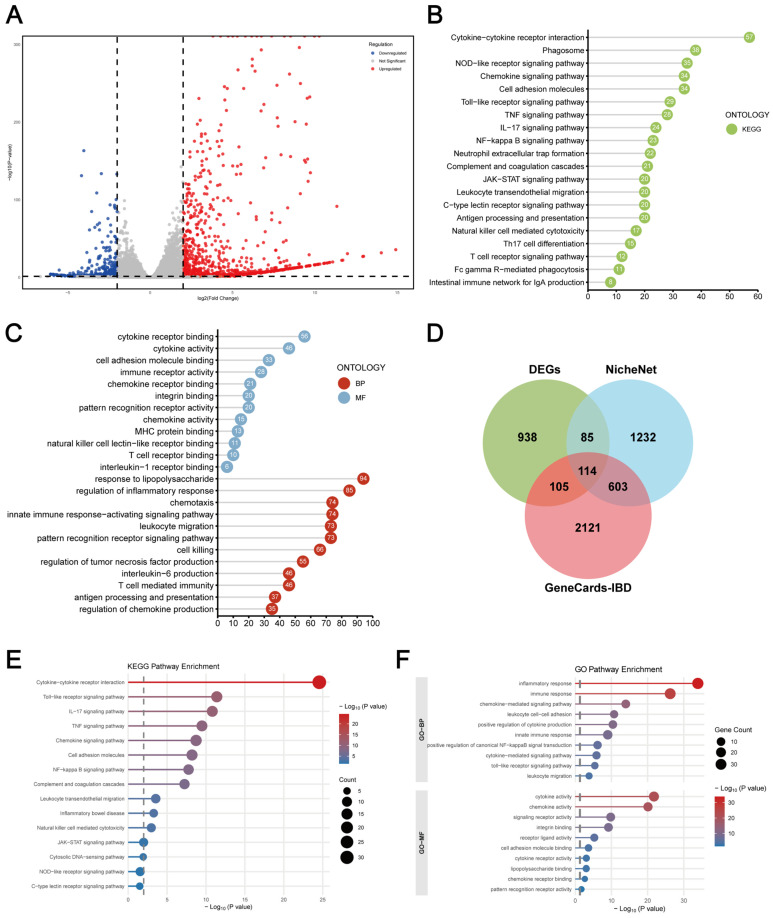
Transcriptomic analysis of intestinal organoids based on the co-culture system showed that BBR regulates macrophage–epithelial interactions. Overall, 50 μg/mL of LPS and 1 μM of BBR were applied to the intestinal organoids in the lower chamber of the co-culture system and treated for 24 h. Extracted RNA from the intestinal organoids was collected and analysed for transcriptomics. The 1242 DEGs obtained from transcriptomics analysis were mapped as volcano plots (**A**) and subjected to KEGG (**B**) and GO (**C**) enrichment analyses. A Venn diagram analysis (**D**) was performed on the set of DEGs obtained from transcriptomics, the set of intercellular communication genes obtained from the NicheNet analysis tool, and the set of IBD-related genes based on the GeneCards database. KEGG (**E**) and GO (**F**) enrichment analyses were performed for the 114 intersecting genes in the centre of the Venn diagram. The experiment was performed in triplicate and repeated 3–4 times.

After BBR addition, 1242 DEGs were screened (Figure 7A), KEGG (Figure 7B) and GO (Figure 7C) pathway enrichment analysis confirmed the significant involvement of cytokine–cytokine receptor interaction and MAPK signalling pathway in the presence of RAW264.7 cells (*p* < 0.05, Foldchange > 2). Overlap analysis (Figure 7D) of DEPs, NicheNet, and IBD gene set identified 114 DEPs involved in inflammatory process, cell migration, proliferation, LPS response, TNF regulation, and cytokine/chemokine production. KEGG (Figure 7E) and GO (Figure 7E) analysis linked these DEPs to cytokine–cytokine receptor interaction. In addition, key pathways targeted by BBR [13,14,15] including TNF signalling, PI3K-AKT signalling, MAPK, NF-κB and Janus kinase–signal transducer and activator of transcription (JAK-STAT) pathways, exhibited significant alterations upon RAW cell addition. These findings suggested that in the macrophage–intestinal organoid co-culture model more effectively simulates inflammatory epithelial injury and elucidates the cell–cell interaction mechanisms modulated by BBR.

## 3. Discussion

In the present study, we employed a co-culture system of murine intestinal organoids and RAW264.7 macrophages to elucidate the anti-inflammatory effects and mechanisms of BBR in intestinal inflammation. While previous studies have identified the anti-inflammatory properties of BBR [8], our findings further demonstrate its capacity to modulate epithelial–macrophage interactions within this organoid-based co-culture model.

Since the establishment of intestinal organoids by Hans Clevers’ team in 2009 [21], organoids serve as ideal epithelial barrier models for studying intestinal disease pathogenesis and drug sensitivity in vitro. Using this system, we characterised the protective effects of BBR against LPS-induced inflammation. Although 50 µg/mL LPS caused no significant morphological alterations after 24 h treatment, it reduced cell viability and organoid budding ratio, increased intestinal permeability, and significantly upregulated inflammatory genes. The effect of LPS may be related to the limited expression of toll-like receptor 4 (TLR4) in intestinal organoid [26]. In the previously reported LPS-induced sepsis model, LPS at concentrations above 150 µg/mL was required to induce significant growth inhibition and tissue injury in intestinal organoids [27]. Notably, BBR treatment effectively antagonised LPS-induced increase in inflammatory genes and restored the viability and proliferation of intestinal organoids.

The intestinal epithelium, located between the lamina propria immune cells and intestinal lumen, not only serves as the primary barrier against luminal contents, but also responsible for communicating with immune cells. The epithelial cells modulate immune responses, while simultaneously responding to cytokine signals from immune cells. This epithelial–immune interaction is pivotal for maintaining intestinal mucosal homeostasis. However, the influence of distinct immune cell subsets on the intestinal epithelial barrier under either homeostasis or pathological conditions remains poorly understood, primarily due to the inherent absence of immune components in intestinal organoid cultures. To address this limitation, emerging co-culture systems have been developed [28]. The intestinal organoids derived from induced pluripotent stem cells (iPSCs) or adult stem cells are co-cultured with key immune cells including innate immune cells (e.g., macrophages or dendritic cells) and adaptive immune cells (e.g., T cells) via diverse approaches. The immune cells can be directly co-cultured with organoid in Matrigel, 3D bioprinting structure, or via microinjection [29,30,31], as well as the indirect system using Transwell system or microfluidic chips [32,33]. Among these immune populations, macrophages represent a predominant focus due to their intestinal abundance and well-established role as pathogenic drivers in inflammatory diseases such as IBD. Co-culture studies employ bone marrow-derived macrophages (BMDMs), peripheral blood mononuclear cell (PBMC)-derived macrophages, or macrophage cell lines and demonstrate bidirectional regulations between macrophages and organoids: intestinal organoids influence macrophage polarisation and chemotactic migration, while macrophages exert significant effects on organoid morphology, differentiation, proliferation, and wound injury repair functions [34]. In this study, we employed a Transwell co-culture system to model macrophage–epithelial interactions. Intestinal organoids were cultured in the lower chamber, while macrophages were seeded in the upper chamber. LPS and BBR were added to the organoid culture medium to simulate inflammatory conditions and therapeutic intervention, respectively.

In the Transwell co-culture system, LPS stimulation significantly increased the release of IL-6, CXCL1 and MIF. It correlated with enhanced macrophage infiltration that exacerbating epithelial barrier damage. Immunofluorescence staining and Western blot assay demonstrated the altered ZO-1 expression and distribution in intestinal organoids after LPS treatment, characterised by reduced protein expression and discontinuous patterns. Adherens junction (AJ) proteins E-cadherin, claudin-1, and occludin remained unaffected. These data were consistent with prior findings that BBR prevents the redistribution of ZO-1 from the apical TJ complex to the cytoplasmic compartment of colon epithelial cells and repair the intestinal barrier [35]. Furthermore, flow cytometric analysis further revealed BBR-mediated suppression of M1 macrophage polarisation. The identified mechanism of BBR on M1 macrophage was related to the inhibition of PI3K/AKT/mTOR and MAPK/NF-κB pathways, as reported [36,37]. Collectively, these data indicate BBR targets both epithelial barrier integrity and macrophage function.

To clarify the mechanism of BBR effects on organoids in co-culture system, we conducted proteomic analysis and identified DEPs enriched in ROS metabolism, PI3K-Akt signalling, tight junction function, and innate immune response, which was consistent with previous reports [38]. Subsequently, we employed the NicheNet database to map intercellular communication networks and the GeneCards database to identify proteins associated with IBD pathogenesis. Intersection analysis of these datasets identified ten candidate proteins relevant to epithelial–macrophage crosstalk and IBD pathology. Among these were tight junction protein CLDN7 and critical epithelial–macrophage mediators including IL-1RN, PTGS2 and MIF. PTGS2, also known as cyclooxygenase-2, is a key inflammatory enzyme driving intestinal inflammation, mucosal injury, and immune dysregulation in IBD [39] and also identified as a target of BBR against LPS-induced intestinal damage [40]. MIF is a multifunctional immunomodulator that promotes macrophage chemotaxis and exacerbates inflammatory injury. Its elevated expression was identified in IBD patients [41,42]. Importantly, our follow-up validation demonstrated that BBR significantly suppressed the release of pro-inflammatory chemokines MIF and CXCL1. Furthermore, molecular docking studies established a potential interaction between BBR and MIF protein. These findings collectively reveal that BBR intervention effectively inhibited the expression and release of inflammatory mediators in organoids, thus decreasing the polarisation and infiltration of macrophages to the M1 subtype, suggesting that BBR effectively disrupting this pathological macrophage–epithelial interactions, thereby effectively reducing intestinal inflammation.

Notably, phenotypic and transcriptomic analyses revealed that LPS significantly amplifies inflammatory responses in the co-culture system, likely through the regulation of multiple innate immune and inflammatory response pathways. This observation aligns with the previous literature demonstrating that macrophages markedly elevate cytokine production under inflammatory conditions, subsequently triggering intestinal organoids to release both pro-/anti-inflammatory mediators that collectively amplify mucosal immune activation and drive tissue fibrosis [43]. Crucially, in the presence of macrophages, a significant enrichment of IBD related cytokine–cytokine receptor interactions were observed after BBR treatment. Collectively, these findings demonstrate that the macrophage–organoid co-culture represents a physiologically relevant model for investigating intestinal mucosal inflammation and developing cell interaction-targeted therapeutics.

## 4. Materials and Methods

### 4.1. Reagents and Chemicals

BBR was purchased from the China Institute for Food and Drug Control and dissolved dimethyl sulfoxide (DMSO). LPS-B5 is a preparation of smooth (S)-form lipopolysaccharide from the Gram-negative bacteria *E. coli* O55:B5, purchased from InvivoGen (San Diego, CA, USA). Both reagents were diluted in culture medium to the appropriate experimental concentration.

### 4.2. Mice

C57BL/6 mice (6–8 weeks old, Specific Pathogen Free) were purchased from Charles River (Beijing, China). The experiment was performed in accordance with the relevant regulations of the Laboratory Animal Welfare and Ethics Committee of Beijing University of Technology (Approval No. HS202409002). Mice aged 6 to 8 weeks were used for crypt isolation and organoid culture.

### 4.3. Cell Culture

Mouse macrophage cell line *RAW264.7* were purchased from American Type Culture Collection (ATCC, Manassas, VA, USA) and maintained in DMEM high glucose medium (Gibco, Grand Island, NY, USA) containing 1% penicillin-streptomycin and 10% foetal bovine serum (FBS, Gibco, Grand Island, NY, USA). Cells were incubated at 37 °C in an incubator with 5% CO_2_ and subcultured every 3–4 days upon reaching 80–90% confluence.

### 4.4. Crypt Isolation and Organoid Culture

Crypts were isolated and cultured with minor modifications according to the manufacturer’s instructions. In brief, approximately 20 cm of murine small intestine, proximal to the gastric pylorus, was excised and dissected into 2 mm fragments. After extensive washing, tissues were digested in Gentle Cell Dissociation Reagent (STEMCELL, Vancouver, BC, Canada) for 15 min at room temperature (RT). Crypts were then filtered through a 70 μm strainer and collected for quantification. Approximately 200 crypts were embedded in 50 μL Matrigel (Corning, NY, USA) and seeded into 24-well plates. After polymerization, 500 μL of Advanced DMEM/F12 medium (Gibco, Grand Island, NY, USA) supplemented with 1× GlutaMAX (Gibco, Grand Island, NY, USA), 10 mM HEPES (Gibco, Grand Island, NY, USA), 1% penicillin/streptomycin, 1× N2 supplement (Gibco, Grand Island, NY, USA), 1× B27 supplement (Gibco, Grand Island, NY, USA), 1 mM N-Acetylcysteine (Sigma-Aldrich, St. Louis, MO, USA), 50 ng/mL EGF (PeproTech, Rocky Hill, NJ, USA), 100 ng/mL Noggin (Novoprotein, Suzhou, China), 500 ng/mL R-spondin-1 (Novoprotein, Suzhou, China), and 3 μM CHIR99021 (Sigma-Aldrich, St. Louis, MO, USA) was added. The culture medium was changed every 2–3 days, and organoids were passaged every 5–7 days. The budding rate of organoids was determined by counting at least 150 organoids per condition using ImageJ software (v2.14.0).

### 4.5. Cell Viability Assay

Cell viability was assessed using the CellTiter-Lumi™ Luminescent 3D Cell Viability Assay Kit (Beyotime, Shanghai, China) according to the manufacturer’s instructions. Murine intestinal organoids embedded in Matrigel were seeded in 96-well white opaque plates (Corning, NY, USA). After 3 days of culture, the medium was replaced with differentiation medium prior to treatment with BBR (0.3–3 μM) or LPS (50 μg/mL). Following 24 h incubation, an equal volume of CellTiter-Lumi™ reagent was added per well. Luminescence was recorded using an Enspire Multimode Plate Reader (PerkinElmer, Waltham, MA, USA).

### 4.6. EdU Cell Proliferation Assay

Cell proliferation assays were performed using the BeyoClick™ EdU-594 Cell Proliferation Assay Kit (Beyotime, Shanghai, China). Briefly, after BBR treatment for 24 h, EdU (10 μM) was introduced for a 3 h incubation. Organoids were then fixed, permeabilised, and processed for EdU Click reaction. Organoids were co-stained with Hoechst 33342 (Beyotime, Shanghai, China). Images were captured and analysed using Operetta High-content imaging system (Perkin Elmer, Waltham, MA, USA).

### 4.7. FITC-Dextran Permeability Assay

The intestinal epithelia barrier function was evaluated by measuring FITC dextran permeability. Organoids were cultured with 4 kDa FITC-dextran (5 μg/mL, MedChemExpress, Monmouth Junction, NJ, USA) for 1 h. After PBS washing, imaging was performed using an Operetta High-content imaging analysis system (Perkin Elmer, Waltham, MA, USA).

### 4.8. Transwell Co-Culture of Murine Intestinal Organoid and RAW264.7 Cells

Mouse macrophage RAW264.7 cells and murine intestinal organoids were co-cultured using Transwell plates (8 μm pore, Corning, NY, USA). Organoids embedded in Matrigel were seeded in the lower chamber and pre-cultured for 3 days. Then, RAW264.7 cells were added at a density of 2 × 10^5^/mL to the upper chamber. The culture medium in the lower chamber was replaced with differentiation medium containing BBR (0.3–3 μM) or LPS (50 μg/mL). After 24 h co-culture, medium was collected for cytokine ELISA analysis, organoids were harvested for RNA and protein analysis, and RAW264.7 cells were assessed via Transwell migration assays and flow cytometry for M1 polarisation analysis.

### 4.9. Transwell Migration Assay

Following the 24 h co-culture of RAW264.7 cells and murine intestinal organoids, RAW264.7 cells on the upper membrane surface were removed using cotton-tipped applicators. The inserts were subsequently fixed in 4% paraformaldehyde for 20 min at room temperature, and stained with 0.1% crystal violet for 10 min. After three washes of distilled water, membranes were excised and mounted on glass slides. Migrated cells were visualised and counted in five randomly selected fields per membrane using ImageJ software. The CXCR2 inhibitor SB225002 (50 nM, TargetMol, Boston, MA, USA) was used as positive control.

### 4.10. Flow Cytometry Analysis

Flow cytometry was used to analyse the expression of CD80 and CD86 in RAW264.7 cells in the co-culture system. RAW264.7 cells were collected from the Transwell upper chamber using TrypLE (Gibco, Grand Island, NY, USA) and filtered through 40 μm cell strainers. Cells were blocked with TruStain FcX (anti-mouse CD16/32, BioLegend, San Diego, CA, USA) for 10 min, followed by staining with PE anti-mouse CD86 (BioLegend, San Diego, CA, USA) and PerCP/Cy5.5 anti-mouse CD80 antibody (BioLegend, San Diego, CA, USA) for 30 min. Flow cytometry analysis was performed using FACSCanto II flow cytometer (BD Bioscience, Franklin Lakes, NJ, USA) and data were analysed using FlowJo software (v10.8.1).

### 4.11. Immunofluorescence

Organoids were fixed in 4% paraformaldehyde for 30 min at room temperature, permeabilized using 0.3% Triton X-100 and 1% Bovine Serum Albumin (BSA), and blocked with buffer containing 0.3% Triton X-100, 1% BSA, 2% normal goat serum, and 1% gelatin. Organoids were stained separately with rabbit anti-mouse ZO-1 antibody (PeproTech, Rocky Hill, NJ, USA) and rabbit anti-mouse E-cadherin antibody (PeproTech, Rocky Hill, NJ, USA) overnight at 4 °C, followed by incubation with DyLight 488-labelled goat anti-rabbit secondary antibody for 2 h at room temperature. Imaging was performed using confocal laser scanning microscope (LSM980, Zeiss, Oberkochen, Germany).

### 4.12. Western Blot Assay

Organoids harvested from Matrigel were lysed using radioimmunoprecipitation assay (RIPA) lysis buffer (Solarbio, Beijing, China) containing protease inhibitor. Total protein concentration was quantified using a bicinchoninic acid protein assay kit (Solarbio, Beijing, China). Sodium dodecyl sulphate-polyacrylamide gel electrophoresis (SDS-PAGE) gels were prepared with the Rapid Protein Gel Kit (Dakewe Biotech, Shenzhen, China), and protein samples were separated by electrophoresis prior to transfer to polyvinylidene difluoride (PVDF) membranes. After blocking using 5% non-fat milk in PBS containing 0.01% Tween 20, membranes were incubated separately with goat anti-mouse ZO-1 antibody (PeproTech, Rocky Hill, NJ, USA), goat anti-mouse E-cadherin antibody (PeproTech, Rocky Hill, NJ, USA), and goat anti-mouse claudin-1 antibody, goat anti-mouse occludin antibody (PeproTech, Rocky Hill, NJ, USA) overnight at 4 °C, and then with horseradish peroxidase (HRP)-conjugated donkey anti-goat secondary antibody for 2 h at room temperature. Protein detection was performed using an enhanced chemiluminescence system (Beyotime, Shanghai, China) and visualised with a Tanon 5200 multi-imaging system (Tanon Science & Technology, Shanghai, China). The band grayscale values were quantified with ImageJ with glyceraldehyde-3-phosphate dehydrogenase (GAPDH) as the internal control.

### 4.13. Enzyme-Linked Immunosorbent Assay (ELISA)

Supernatants from the lower chamber of murine intestinal organoid cultures were collected and analysed for pro-inflammatory cytokines and chemokines using ELISA kits for mouse CXCL1 (BioLegend, San Diego, CA, USA), mouse CXCL5 (PeproTech, Rocky Hill, NJ, USA), mouse IL-6 (BioLegend, San Diego, CA, USA), mouse MCP-1 (BioLegend, San Diego, CA, USA), and mouse MIF (PeproTech, Rocky Hill, NJ, USA) according to the manufacturer’s instructions.

### 4.14. Real-Time Quantitative PCR Analysis

Total RNA was extracted from murine intestinal organoids using TRIzol™ reagent (Thermo Fisher Scientific, Waltham, MA, USA) and then reverse-transcribed to cDNA with NovoScript^®^ Plus All-in-one 1st Strand cDNA Synthesis SuperMix (Novoprotein, Suzhou, China). Quantitative PCR was subsequently performed using iTaq Universal SYBR Green Supermix (Bio-Rad Laboratories, Hercules, CA, USA) on the ViiA 7 Real-Time PCR System (Applied Biosystems, Foster City, CA, USA). Primer sequences are listed in Table 1.

### 4.15. Proteomic Analysis

Murine intestinal organoids were analysed by a Thermo Orbitrap Eclipse™ Mass Spectrometer in conjunction with a Thermo-Dionex Ultimate NCS-3500RS NANO Liquid Chromatography system. Peptides were separated on a capillary analytical column (New Objective, Littleton, MA, USA, 25 cm length × 75 µm internal diameter, 10 µm emitter tip) connected to the SPE column via a PEEK union (Valco instruments, Houston, TX, USA). There was a flow rate of 0.3 µL/min. The mobile phase A was composed of 0.1% formic acid, while mobile phase B comprised 80% acetonitrile and 0.1% formic acid. Mass spectrometry analysis was performed using data-independent acquisition (DIA), with the full width at half maximum (FWHM) set to 18 s. Full-scan mass spectra were obtained using an Orbitrap using Xcalibur 3.0 software; proteomic data were collected and analysed by DIA-NN (v1.8.1). Data were imputed and normalised for missing values, followed by a *t*-test to calculate fold change and *p* value for each protein. For the data with *p* < 0.05 and logFC > 0.585, KEGG, GO and GSEA analyses were performed using R packages “clusterProfiler (v4.14.4)” and “enrichplot (v1.26.6)” in R software (v.4.0.5). PPI networks were constructed using the STRING database and Cytoscape (v3.7.2). Intercellular communication gene sets were obtained using NicheNet (v2.2.1); IBD-related gene sets were obtained using the GeneCards database (https://www.genecards.org/; accessed on 5 July 2025). Venn diagrams were generated using Adobe Illustrator CC 2018. MIF docking analysis was performed using AutoDock Vina (v1.2.5).

### 4.16. RNA Sequencing (RNA-Seq) Analysis

Total RNA was isolated from murine intestinal organoid using TRIzol™ reagent and subjected to transcriptome sequencing on a DNBSEQ-T7 platform (IgeneCode Biotech, Beijing, China). Differentially expressed genes (DEGs; *p* < 0.05, logFC > 2) were subjected to KEGG, GO and GSEA analyses using R packages “clusterProfiler (v4.14.4)” and “enrichplot (v1.26.6)” in R software (v.4.0.5). GSEA was performed using the M2: curated gene sets (immunologic signatures) from Molecular Signatures Database (MSigDB). Intercellular communication gene sets were obtained using NicheNet (v2.2.1); IBD-related gene sets were obtained using the GeneCards database (https://www.genecards.org/; accessed on 5 July 2025). Venn diagrams were generated using Adobe Illustrator CC 2018.

### 4.17. Statistical Analysis

Data were presented as mean + standard deviation (SD). Statistical analyses were performed with GraphPad Prism 9 software (GraphPad Software Inc. v9.5.0) using one-way analysis of variance (ANOVA), and *p* < 0.05 was considered statistically significant. The mass spectrometry proteomics data have been deposited to the ProteomeXchange Consortium (https://proteomecentral.proteomexchange.org/; accessed on 22 July 2025) via the iProX partner repository [44,45] with the dataset identifier PXD066396. The RNA-seq raw sequence data reported in this paper have been deposited in the Genome Sequence Archive [46,47] (Genomics, Proteomics & Bioinformatics 2021) in the National Genomics Data Center (Nucleic Acids Res 2022), the China National Center for Bioinformation/Beijing Institute of Genomics, and the Chinese Academy of Sciences (GSA: CRA028387), which are publicly accessible at https://ngdc.cncb.ac.cn/gsa/ (accessed on 29 July 2025).

## 5. Conclusions

In summary, we employed a murine intestinal organoid–macrophage co-culture model to explore the anti-inflammatory mechanism of BBR. The Transwell co-culture model demonstrated more evident inflammation and tissue damage compared to organoid monoculture. Within this model, BBR exerted dual protective effects on attenuating M1 macrophage polarisation and restoring epithelial integrity. Critically, by disrupting pathological macrophage–epithelial interaction through the inhibition of pro-inflammatory chemokines including MIF, BBR effectively reduced immune cell infiltration, thereby attenuating intestinal inflammation.

## Figures and Tables

**Table 1:** Names and sequences of primers used for RT-PCR.

Name	Sequence
*cxcl1* F	GCACCCAAACCGAAGTCATAG
*cxcl1* R	CGTTACTTGGGGACACCTTTTAG
*cxcl2* F	CAGACAGAAGTCATAGCCACTC
*cxcl2* R	CTCTTTGGTTCTTCCGTTGAGG
*cxcl5* F	TGCCCTACGGTGGAAGTCATA
*cxcl5* R	TGCATTCCGCTTAGCTTTCTTT
*cox2* F	TGCACTATGGTTACAAAAGCTGG
*cox2* R	TCAGGAAGCTCCTTATTTCCCTT
*crp* F	TTCCCAAGGAGTCAGATACTTCC
*crp* R	TCAGAGCAGTGTAGAAATGGAGA
*il1b* F	GAAATGCCACCTTTTGACAGTG
*il1b* R	TGGATGCTCTCATCAGGACAG
*gapdh* F	AGGTCGGTGTGAACGGATTTG
*gapdh* R	TGTAGACCATGTAGTTGAGGTCA

## Data Availability

The mass spectrometry proteomics data have been deposited to the ProteomeXchange Consortium (https://proteomecentral.proteomexchange.org/; accessed on 22 July 2025) via the iProX partner repository [44,45] with the dataset identifier PXD066396. The RNA-seq raw sequence data reported in this paper have been deposited in the Genome Sequence Archive [46,47] (Genomics, Proteomics & Bioinformatics 2021) in National Genomics Data Center (Nucleic Acids Res 2022), China National Center for Bioinformation/Beijing Institute of Genomics, Chinese Academy of Sciences (GSA: CRA028387) that are publicly accessible at https://ngdc.cncb.ac.cn/gsa/ (accessed on 29 July 2025).

## Supplementary Materials

The supporting information can be downloaded at: https://www.mdpi.com/article/10.3390/ijms262010161/s1.
