# Supplementary material for: Berberine Alleviates Intestinal Inflammation by Disrupting Pathological Macrophage–Epithelial Crosstalk in Macrophage–Organoid Co-Culture Model
Source: Int J Mol Sci. 2025 Oct 19;26(20):10161. doi: 10.3390/ijms262010161 (PMC12563409; doi:10.3390/ijms262010161)
Supplement: Supplementary file 1 [file ijms-26-10161-s001.zip › ijms-3895182-supplementary.pdf]

Supplementary material

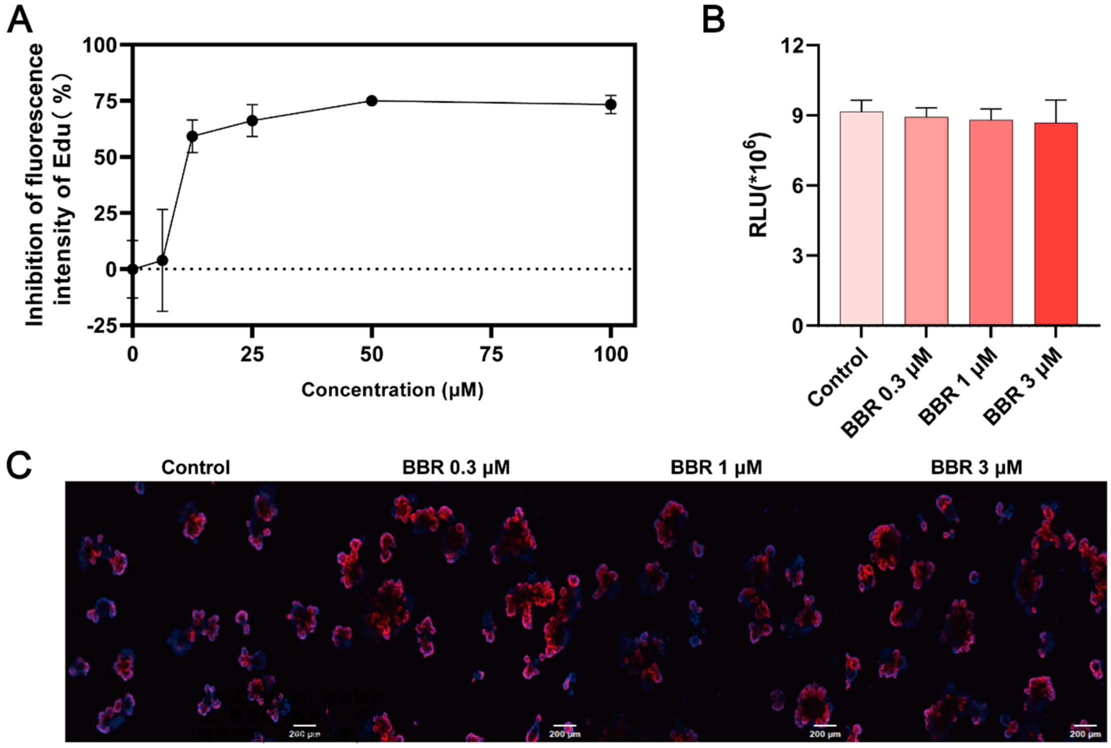

Figure S1 Toxicity test of BBR on intestinal organoids. (A) 0-100 μM BBR was applied to intestinal organoids and treated for 24 h. Proliferation of intestinal organoids was detected using the EdU cell proliferation assay. 0.3-3 μM BBR was applied to intestinal organoids and treated for 24 h. (B) Cell viability of intestinal organoids was detected using the CellTiter-Glo assay. (C) Proliferation of intestinal organoids was detected using the EdU cell proliferation assay and immunofluorescence staining, and imaged using the Operetta High-content imaging analysis system, scale bar, 200 μm. The data presented represent the pooled results from two or three independent experiments.

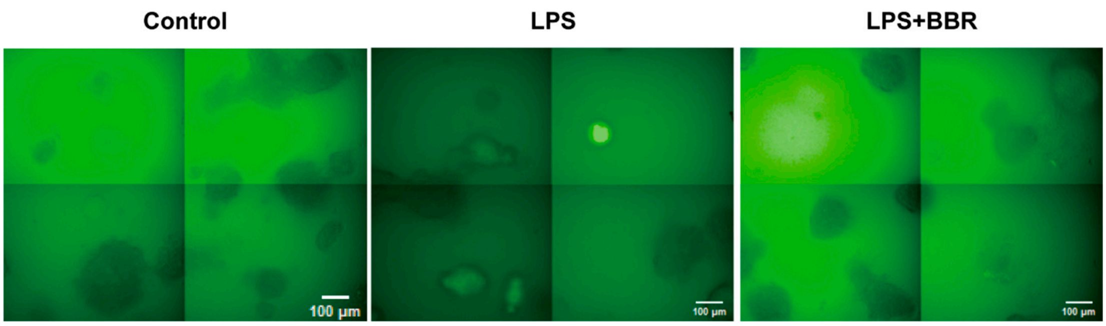

Figure S2 BBR has a reparative effect on inflammation-induced epithelial damage. Intestinal organoids were treated with 50 μg/ml lipopolysaccharide (LPS) and 1 μM BBR for 24 h. The epithelial barrier function was examined using the FITC-dextran permeability assay, and imaging was performed using the Operetta High-content imaging analysis system, scale bar, 100 μm. The experiment was conducted three times.

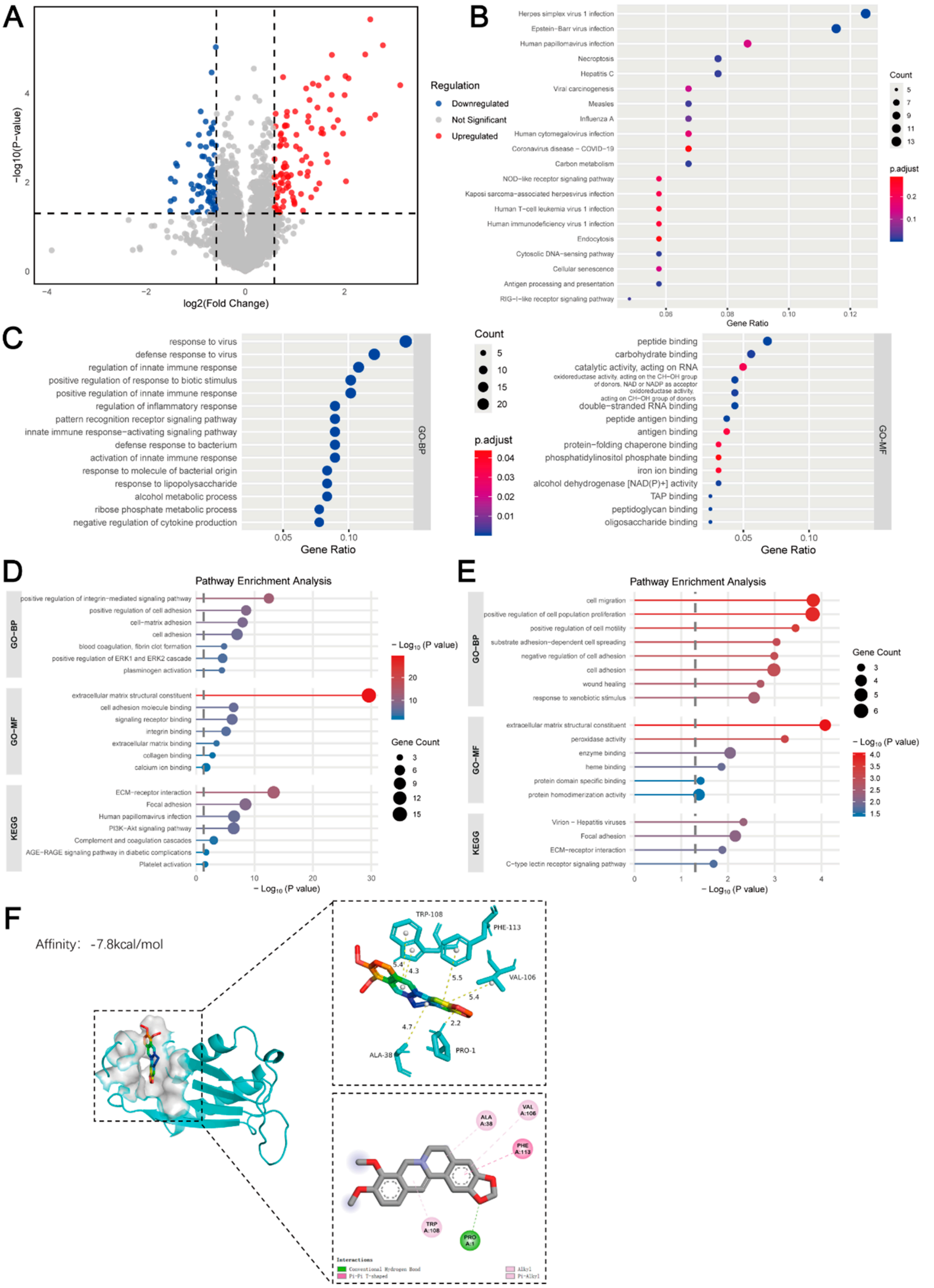

Figure S3 Proteomic analysis of the effect of BBR on intestinal organoids in inflammation. 50 μg/ml LPS and 1 μM BBR were applied to intestinal organoids and treated for 24 h. The proteins were collected for proteomic analysis. The DEPs obtained by comparing the LPS and control treatment groups were plotted as volcano plots (A) and analysed for KEGG (B) and GO (C) enrichment. Overlap analyses were performed on DEPs obtained from proteomics analyses, on the set of intercellular communication genes obtained from the NicheNet analysis tool, and on the set of IBD-related genes based on the GeneCards gene library. GO and KEGG

enrichment analyses were performed for the 25 intersecting genes in the DEPs and NicheNet gene sets (D), GO and KEGG enrichment analyses were performed for the 30 intersecting genes in the DEPs and IBD gene sets (E), and molecular docking analyses were performed for MIFs in the intersecting sets of the three gene sets (F). The experiment was performed in triplicate and repeated 3–4 times.
